# Transcriptomic Insights into the Synergistic Mechanism of Spinosad–Indoxacarb Mixtures Against *Cnaphalocrocis medinalis*

**DOI:** 10.3390/insects17060598

**Published:** 2026-06-07

**Authors:** Hong-Shuang Li, Meng-Zhen Wang, Ji-Yang Xing, Cong-Fen Gao, Shun-Fan Wu

**Affiliations:** 1 College of Plant Protection, Nanjing Agricultural University, Nanjing 210095, China; vaexxxx2022@163.com (H.-S.L.); 13965261983@163.com (M.-Z.W.); 2023202080@stu.njau.edu.cn (J.-Y.X.); 2 College of Plant Protection, Sanya Institute of Nanjing Agricultural University, Sanya 572025, China; 3 State Key Laboratory of Agricultural and Forestry Biosecurity, Nanjing 210095, China

**Keywords:** *Cnaphalocrocis medinalis*, insecticide mixture, spinosad, indoxacarb, synergistic mechanism, transcriptome

## Abstract

The rice leaffolder, *Cnaphalocrocis medinalis*, has developed widespread resistance to conventional insecticides, severely threatening rice production. Pesticide combination is a critical strategy for enhancing efficacy and delaying resistance evolution. In this study, binary mixtures with distinct modes of action were screened against *C. medinalis* neonates. Bioassays indicated that mixtures of spinosad and indoxacarb exhibited significant synergism, while several other combinations showed antagonism. To elucidate the synergistic mechanism of the optimal 1:1 mixture, comparative transcriptomic analyses were performed. Results revealed a time-dependent dual-phase response. At 6 h, the mixture induced broader upregulation of detoxification genes and activated stress signaling pathways due to competitive enzyme saturation, impairing metabolic clearance and amplifying neurotoxicity. At 24 h, massive transcriptional reprogramming uniquely activated lysosome, autophagy, lipid metabolism, and neurodegenerative disease pathways. This study demonstrates that the synergism arises from early competitive inhibition of detoxification and late overactivation of the autophagy–lysosomal system associated with cellular damage. These findings provide transcriptomic evidence for the dynamic synergistic mechanism and offer a scientific basis for rational resistance management of *C. medinalis*.

## Simple Summary

*Cnaphalocrocis medinalis* has developed strong resistance to many conventional insecticides, threatening rice production. In this study, we tested mixtures of different insecticides and found that combining spinosad with indoxacarb produced a strong synergistic effect—killing the pests more effectively than either insecticide alone. By analyzing gene expression changes, we discovered that the mixture works in two phases: first, it blocks the insect’s detoxification systems, making the toxins last longer; second, it overactivates cellular self-digestion (autophagy) and damages midgut cells, resulting in autophagy–lysosomal activation and cellular damage. These findings help explain how insecticide mixtures can be designed to overcome resistance and provide a scientific basis for more sustainable pest control.

## 1. Introduction

Rice (*Oryza sativa* L.) is a staple food crop sustaining nearly half of the global population, contributing approximately 21% of the world’s caloric intake and underpinning food security for millions of communities worldwide [1]. However, rice production is persistently threatened by biotic stresses, with insect pests causing global yield losses ranging from 24.6% to 40.9% [2]. The rice leaffolder, *Cnaphalocrocis medinalis* (Lepidoptera: Crambidae), a highly destructive migratory pest, is a major contributor to these losses: its larval stages roll rice leaves and feed on mesophyll tissue, directly impairing photosynthetic capacity and reducing grain yield and quality. As a nationally regulated pest in China, *C. medinalis* has experienced frequent outbreaks in major rice-growing regions in recent years, exacerbating threats to local and regional rice production.

Chemical control remains the primary and most rapid strategy for managing acute *C. medinalis* infestations, given the limitations of cultural and biological control in coping with large-scale migratory outbreaks. For decades, diamide insecticides (e.g., chlorantraniliprole) and macrocyclic lactones (e.g., abamectin, emamectin benzoate) have been the mainstay of chemical control programs for *C. medinalis*, valued for their high target efficacy and low non-target toxicity. However, the long-term, repetitive, and single-mode-of-action application of these insecticides has driven the rapid evolution of resistance in field populations of *C. medinalis*. Recent monitoring data have documented >100-fold resistance ratios to diamides in multiple field populations of *C. medinalis* in China, with moderate resistance also emerging to macrocyclic lactones [3,4]. This resistance crisis has created a vicious cycle: reduced control efficacy necessitates higher application rates, which in turn increases environmental contamination, agricultural costs, and further selection pressure for resistance, while also disrupting farmland ecological balance by harming natural enemies and non-target organisms. The escalating resistance of *C. medinalis* to conventional insecticides thus demands the urgent development of sustainable, science-based pest management strategies to restore control efficacy and delay further resistance evolution.

Pesticide compounding (the formulation of binary or multi-component insecticide mixtures) has emerged as a core strategy in modern integrated pest management (IPM) for addressing insecticide resistance [5]. When rationally designed, insecticide mixtures offer multiple ecological and toxicological advantages: they enhance control efficacy through synergistic interactions, reduce selection pressure for resistance by targeting multiple molecular sites, expand the control spectrum to manage concurrent pests, and lower total pesticide input by reducing individual active ingredient dosages [6,7]. For rice pests, successful applications of synergistic mixtures have been reported, including imidacloprid–chlorpyrifos for planthopper and stem borer control and abamectin–pymetrozine for brown planthopper (*Nilaparvata lugens*) management [8]. However, the efficacy of insecticide mixtures is not universal: inappropriate combinations (e.g., compounds with overlapping metabolic pathways or competitive target binding) can result in antagonistic effects, reducing control efficacy and potentially accelerating resistance development [9,10]. This underscores the critical need for empirical screening of mixtures and rigorous evaluation of their combined toxicity using quantitative indices such as the co-toxicity coefficient (CTC).

While formulation screening is essential, understanding the molecular mechanisms underlying synergistic or antagonistic interactions is equally critical for the rational design and deployment of insecticide mixtures. Synergism in pesticide mixtures typically arises from two primary types of interactions: toxicokinetic (interference with absorption, distribution, metabolism, or excretion of active ingredients) and toxicodynamic (convergence or amplification of effects at molecular targets, signaling pathways, or physiological processes) [11]. For example, inhibition of cytochrome P450 monooxygenases (CYP450s) or esterases by one compound can impair the metabolic detoxification of another, leading to toxic accumulation at target sites [12]; similarly, convergent activation of stress signaling pathways or cellular damage pathways by distinct insecticides can overwhelm pest defensive systems, leading to enhanced mortality [13]. Until recently, studies on insecticide mixtures for *C. medinalis* have been limited to phenotypic toxicity evaluation and CTC calculation, with little known about the molecular and cellular mechanisms driving synergistic effects. This knowledge gap severely restricts the optimization of mixture formulations and the prediction of long-term resistance risks associated with their field use.

Transcriptomic analysis has emerged as a powerful tool for unraveling the molecular mechanisms of pesticide action and synergism, enabling the identification of differentially expressed genes (DEGs), perturbed signaling pathways, and cellular responses to single and combined pesticide exposure [14]. By comparing transcriptomic profiles of pests exposed to insecticide mixtures versus single active ingredients, researchers can identify unique molecular responses that underpin synergism, such as disrupted detoxification systems, overactivated stress pathways, or cellular damage. For *C. medinalis*, transcriptomic approaches have yet to be applied to dissect the synergistic mechanisms of insecticide mixtures, representing a key research gap.

In this study, we first screened a panel of binary insecticide mixtures with distinct modes of action against *C. medinalis* neonates using the rice seedling dip method, with CTC analysis to identify synergistic combinations. We then focused on the promising synergistic mixture—spinosad and indoxacarb—and performed comparative transcriptomic analysis of *C. medinalis* larvae exposed to spinosad alone, indoxacarb alone, or their 1:1 mixture at 6 h and 24 h post-exposure. Both spinosad and indoxacarb are commonly used field insecticides, and their mixture has been officially registered in China for controlling *C. medinalis* in rice. Therefore, investigating the synergistic mechanism of this combination has direct practical implications and significant field application value. Our objectives were to: (1) identify optimal synergistic ratios of spinosad and indoxacarb for *C. medinalis* control; (2) characterize the time-dependent transcriptomic responses of *C. medinalis* to single versus combined insecticide exposure; (3) unravel the molecular and cellular mechanisms driving the synergistic effect of the spinosad–indoxacarb mixture; and (4) identify potential molecular markers for resistance monitoring and rational formulation design. We hypothesized that the synergism of spinosad and indoxacarb arises from a dynamic, two-stage response: early competitive inhibition of detoxification systems, followed by late activation of cellular damage pathways. The findings of this study provide the first transcriptomic evidence for the synergistic mechanism of an insecticide mixture in *C. medinalis*, and offer a scientific framework for the development of sustainable resistance management strategies for this key rice pest.

## 2. Materials and Methods

### 2.1. Insects

Field populations of *Cnaphalocrocis medinalis* were collected from rice-growing regions in Guangdong and Jiangsu Provinces of China from May to October during 2024–2025. A susceptible laboratory strain (Cm-S) was originally isolated from rice fields at Jiangpu Farm of Nanjing Agricultural University in September 2010, and has been continuously reared on wheat seedlings under insecticide-free conditions since then. All insect colonies were maintained in a climate-controlled incubator with the following conditions: temperature (27 ± 1) °C, relative humidity 70% ± 10%, and a photoperiod of 16 h light:8 h dark.

### 2.2. Chemicals

All insecticides used in this study were technical-grade raw materials with the following purities and suppliers: abamectin (96.8%, Hebei Veyong Bio-Chemical Co., Ltd., Shijiazhuang, China); emamectin benzoate (95.2%, Shandong Aokun Crop Science Co., Ltd., Jinan, China); spinetoram (98.1%, DowDuPont, Wilmington, DE, USA); indoxacarb (95%, Jiangsu KeweiBang Agrochemical Co., Ltd., Nanjing, China); metaflumizone (97.8%, BASF China Co., Ltd., Shanghai, China); chlorpyrifos (96.5%, Jiangsu Red Sun Co., Ltd., Nanjing, China); methoxyfenozide (95%, Jiangsu Nanjing KeweiBang Agrochemical Co., Ltd., Nanjing, China); cyproflanilide (98%, Nantong Taihe Chemical Co., Ltd., Nantong China); and quinalphos (92.5%, Shenzhen Noposion Agrochemical Co., Ltd., Shenzhen, China). N,N-Dimethylformamide (DMF, analytical grade) was purchased from Guangdong Guanghua Sci-Tech Co., Ltd., Shantou, China, and Triton X-100 (biochemical grade) was obtained from Solarbio Life Sciences Co., Ltd., Beijing, China.

### 2.3. Bioassay

The rice seedling dip method was adopted to determine the toxicity of single insecticides and their binary mixtures to *C. medinalis* neonates, with minor modifications. Briefly, a 0.1% (*v*/*v*) Triton X-100 aqueous solution was prepared as the diluent and thoroughly stirred to ensure homogeneity. Stock solutions of each technical-grade insecticide were prepared with DMF, and serially two-fold diluted with the 0.1% Triton X-100 diluent to obtain 5–6 test concentrations for each compound/mixture. The control group was treated with 0.1% Triton X-100 aqueous solution without insecticides.

Fifteen-day-old healthy rice seedlings (~20 cm in height) were cultivated in plastic cups (7 cm diameter × 5 cm height), with yellowed or withered leaves removed prior to treatment. Rice seedling leaves were fully immersed in the test solutions (or control solution) for 30 s, then air-dried for approximately 30 min at room temperature and cut into 6 cm long segments (stems excluded). Five layers of circular filter paper (7 cm diameter) were placed in sterile Petri dishes (7 cm diameter), and 1.8–2.2 mL of sterile water was added to moisten the filter paper (no free water on the surface). Eight to ten treated rice leaf segments were placed flat in each Petri dish.

Ten *C. medinalis* neonates (collected via the light-induced silk suspension method) were gently transferred onto the leaf segments using a fine soft brush for each replicate. Each test concentration was set with four biological replicates. Petri dishes were covered with two layers of damp black cloth (10 cm × 10 cm, wet side facing upward to prevent larval drowning) and sealed with lids to avoid larval escape. All Petri dishes were incubated under the standard rearing conditions (27 ± 1 °C, 70% ± 10% RH, 16:8 L:D). Larval mortality was recorded at 72 h post-treatment; larvae that failed to right themselves or crawl upon gentle prodding with a fine needle were considered dead.

### 2.4. Transcriptome Sample Preparation

Neonates of the susceptible *C. medinalis* strain (Cm-S) were treated with three test solutions (0.05 mg/L spinosad (SP), 0.05 mg/L indoxacarb (IND), and 0.05 mg/L spinosad–indoxacarb 1:1 mixture (SI)) and the control solution (0.1% Triton X-100 aqueous solution), following the same rice seedling dip method described in 2.3. Treated whole larvae were collected at 6 h and 24 h post-exposure, respectively. Each treatment group (SP, IND, SI) and the control group (CK) at each time point were set with four biological replicates, with approximately 50 larvae per replicate. All collected larval samples were immediately flash-frozen in liquid nitrogen and stored at −80 °C until RNA extraction, resulting in a total of 32 transcriptome samples (4 groups × 2 time points × 4 replicates).

### 2.5. Transcriptome Sequencing and Differential Gene Expression Analysis

Total RNA was extracted from the frozen larval samples using the TRIzol^®^ Reagent (Invitrogen, Carlsbad, CA, USA) following the manufacturer’s protocol. RNA purity, concentration, and integrity were evaluated using a NanoDrop 2000 spectrophotometer (Thermo Fisher Scientific, Waltham, MA, USA), a Qubit^®^ 3.0 Fluorometer (Invitrogen, USA), and an Agilent 2100 Bioanalyzer (Agilent Technologies, Santa Clara, CA, USA), respectively. Only RNA samples with an RNA Integrity Number (RIN) ≥ 8.0 were used for subsequent library construction.

cDNA libraries were constructed using the NEBNext^®^ Ultra™ RNA Library Prep Kit for Illumina^®^ (NEB, Ipswich, MA, USA) following the standard protocol, and the library quality was assessed on the Agilent 2100 Bioanalyzer. High-throughput sequencing was performed on the Illumina NovaSeq 6000 platform (Illumina, San Diego, CA, USA) by Genedenovo Biotechnology Co., Ltd. (Guangzhou, China), generating 150 bp paired-end reads.

Raw sequencing reads were filtered to obtain clean reads by removing low-quality reads (Q-value < 20), reads containing adaptor sequences, reads with unknown bases (N) accounting for more than 10%, and short reads (<50 bp). Clean reads were aligned to the *C. medinalis* reference genome using Hisat2 v2.0.5 software with default parameters. Gene expression levels were quantified as Transcripts Per Million (TPM) reads.

Differential expression analysis between the treatment groups (SP, IND, SI) and the control group (CK) was performed using the DESeq2 R package (v1.16.1). Differentially expressed genes (DEGs) were identified with the screening criteria of |log_2_ fold change (FC)| > 1 and false discovery rate (FDR) < 0.05. Gene Ontology (GO) enrichment analysis and Kyoto Encyclopedia of Genes and Genomes (KEGG) pathway enrichment analysis of DEGs were conducted using the ClusterProfiler R package (v4.10), with the significance threshold set at FDR < 0.05.

### 2.6. Toxicity and Combined Effect Analysis

Toxicity regression equations, slope (b) and its standard error, median lethal concentration (LC_50_) and its 95% confidence limit (95% FL), and chi-square (χ^2^) values of single insecticides and binary mixtures were calculated using POLO-Plus software 2.0 (LeOra Software, Petaluma, CA, USA). Significant differences in toxicity between treatments were determined by whether the 95% confidence intervals of LC_50_ values overlapped (no overlap indicated a significant difference, *p* < 0.05).

The co-toxicity coefficient (CTC) method was used to evaluate the combined toxic effects of binary insecticide mixtures. Insecticide A is spinosad, and insecticide B is indoxacarb. The relative toxicity index (TI), actual toxicity index of the mixture (ATIM), theoretical toxicity index of the mixture (TTIM), and CTC were calculated using the following formulas [15]:

TIA = (LC_50_ of insecticide A/LC_50_ of insecticide A) × 100 = 100

TIB = (LC_50_ of insecticide A/LC_50_ of insecticide B) × 100

ATIM= (LC_50_ of insecticide A/LC_50_ of mixture M) × 100

TTIM = TIA × PA + TIB × PB

CTC = (ATIM/TTIM) × 100

where

TIA: Relative toxicity index of insecticide A;

TIB: Relative toxicity index of insecticide B;

PA: Percentage of insecticide A in the mixture;

PB: Percentage of insecticide B in the mixture;

ATIM: Actual toxicity index of mixture M;

TTIM: Theoretical toxicity index of mixture M;

CTC: Co-toxicity coefficient.

The evaluation criteria for combined effects were as follows: CTC > 120 indicated a synergistic effect; 80 ≤ CTC ≤ 120 indicated an additive effect; CTC < 80 indicated an antagonistic effect.

### 2.7. Statistical Analysis

All experimental data were collated and analyzed using GraphPad Prism 10 software (GraphPad Software, San Diego, CA, USA). Student’s t-test was used for comparisons between two groups, and one-way analysis of variance (ANOVA) followed by Tukey’s Honestly Significant Difference test (HSD) was used for multiple group comparisons. A *p* value < 0.05 was considered statistically significant. All data are presented as mean ± standard error (SE) unless otherwise specified.

## 3. Results

### 3.1. Screening of Synergistic Binary Insecticide Mixtures Against Cnaphalocrocis medinalis Neonates

To identify effective insecticide mixtures for managing *C. medinalis* and delaying resistance evolution, the rice seedling dip method was used to determine the toxicity of six groups of binary mixtures with distinct modes of action, and the co-toxicity coefficient (CTC) method was applied to evaluate their combined toxic effects (Table 1, Table 2, Table 3 and Table 4). For spinosad–indoxacarb, the tested ratios were selected based on commercially registered formulations to ensure practical relevance. For other binary mixtures, the ratios were chosen to cover a range of theoretical interactions, including equimolar and biased combinations, guided by known modes of action, preliminary bioassays, and literature references, in order to detect potential synergistic or antagonistic effects. The results showed significant differences in the interaction types of different insecticide combinations, with only specific ratios exhibiting stable synergistic effects.

Spinetoram-based mixtures exhibited antagonistic effects against *C. medinalis* neonates: the spinetoram–abamectin mixture (1:5) had a CTC of 59.5, and the spinetoram–emamectin benzoate mixture (1:10) had a CTC of 56.85 (both CTC < 80, Table 1). For spinosad–metaflumizone mixtures, the 1:1 ratio showed an additive effect (CTC = 116, 80 ≤ CTC ≤ 120), while the 2:1 ratio exhibited a strong synergistic effect (CTC = 408, CTC > 120, Table 2). Methoxyfenozide–quinalphos mixtures at 1:1 and 1:2 ratios both showed antagonism (CTC = 96.1 and 77.2, respectively, Table 4).

Notably, spinosad–indoxacarb mixtures at multiple ratios consistently displayed significant synergistic effects (all CTC > 120, Table 3). For the susceptible strain (SS), the 1:1 and 2:3 ratios had CTC values of 121 and 123, respectively; for the field population (YZ25), the 1:5 ratio showed a more pronounced synergistic effect with a CTC of 182.1. Based on these results, the spinosad–indoxacarb 1:1 mixture (SI) was selected as the representative synergistic formulation for subsequent transcriptomic analysis to elucidate the underlying molecular mechanism of synergy.

### 3.2. Study on the Combination Mechanism of Spinosad and Indoxacarb

To characterize the transcriptional responses of *C. medinalis* to spinosad (SP), indoxacarb (IND) and their 1:1 mixture (SI), high-throughput transcriptome sequencing was performed on larval samples collected at 6 h and 24 h post-exposure, with four biological replicates for each treatment and control group (CK). Quality control of sequencing data confirmed high reliability and accuracy of the transcriptome dataset (clean read Q30 ≥ 92%, mapping rate to the *C. medinalis* reference genome ≥ 85%).

**Figure 1:**
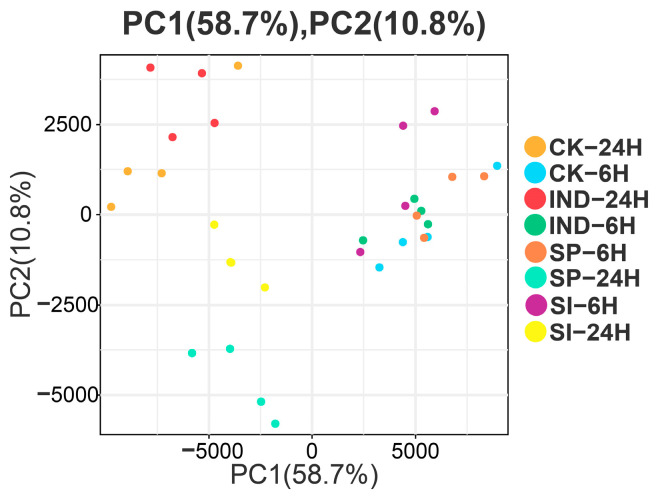
Principal component analysis (PCA) of transcriptome samples under different treatments and time points.

PCA based on FPKM values of all expressed genes showed that PC1 and PC2 explained 58.7% and 10.8% of the total variance, respectively. Samples were clearly separated by treatment and time point: control groups (CK-6h, CK-24h) clustered on the left, single-insecticide treatments (IND, SP) were distributed in the middle, and the spinosad–indoxacarb mixture (SI) samples were located on the right, distinctly separated from both control and single treatments (Figure 1).

**Figure 2:**
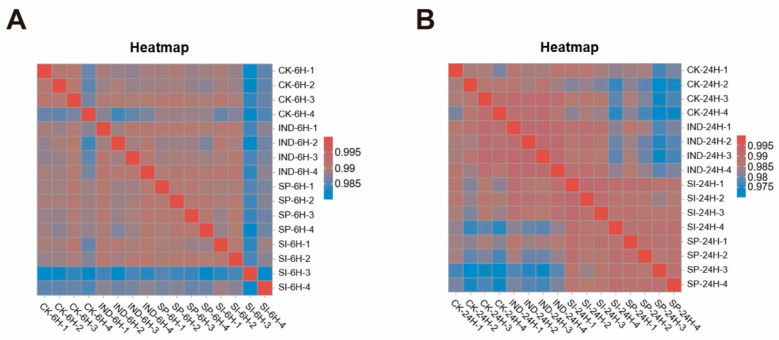
Pearson correlation heatmaps of gene expression among all samples. (**A**) Correlation analysis for samples collected at 6 h post-treatment; (**B**) Correlation analysis for samples collected at 24 h post-treatment. Color gradient from blue to red represents increasing Pearson correlation coefficient values between paired samples.

Sample correlation analysis (Figure 2) revealed that the Pearson correlation coefficients among the four biological replicates of all treatment groups (SP, IND, SI) and CK at both 6 h and 24 h ranged from 0.975 to 0.99, all close to 1. This indicated extremely high repeatability and consistency within groups, and no outlier samples, confirming that the transcriptome data were suitable for subsequent differential gene expression and functional enrichment analyses.

### 3.3. Identification of Differentially Expressed Genes (DEGs) in Response to Single and Combined Insecticide Exposure

Using the stringent screening criteria of |log_2_ fold change (FC)| > 1 and false discovery rate (FDR) < 0.05, DEGs were identified between each insecticide treatment group (SP, IND, SI) and CK at 6 h and 24 h post-exposure (Figure 2). The number of DEGs exhibited temporal dependence and treatment-specific differences, with the SI combination treatment inducing a far more extensive transcriptional reprogramming than the single-agent treatments, especially at the 24 h time point.

At 6 h post-exposure, the number of DEGs in all treatment groups was relatively low: SI treatment induced 42 DEGs (21 upregulated, 21 downregulated), IND treatment induced 33 DEGs (nine upregulated, 24 downregulated), and SP treatment induced only four DEGs (all upregulated). By 24 h post-exposure, the number of DEGs increased dramatically in the SI group, with a total of 687 DEGs identified (605 upregulated, 82 downregulated)—2.5-fold higher than the SP group (273 DEGs, 248 upregulated, 25 downregulated) and 114.5-fold higher than the IND group (only six DEGs, three upregulated, three downregulated). The IND group showed almost no transcriptional response at 24 h, reflecting its relatively weak induction of long-term gene expression changes in *C. medinalis*.

**Figure 3:**
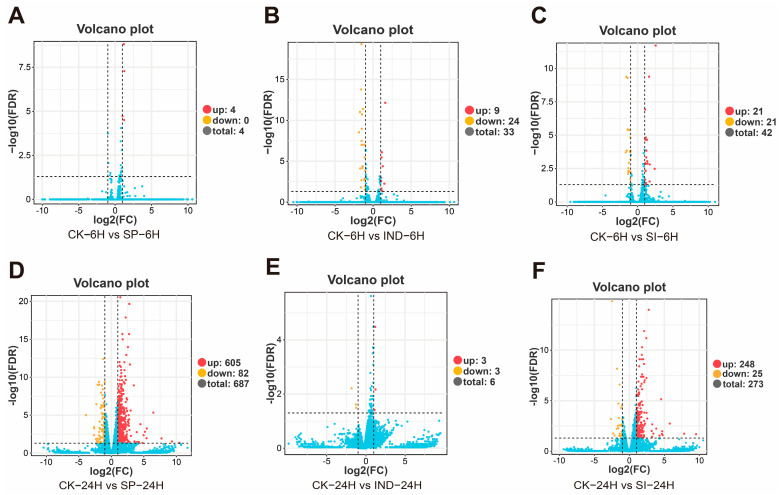
Volcano plots of differentially expressed genes (DEGs) between single-agent and combination treatments at 6 h and 24 h. Red: up-regulated DEGs (FDR < 0.05, log_2_FC > 1); yellow: down-regulated DEGs (FDR < 0.05, log_2_FC < −1); blue: non-significant genes; grey: total DEGs. Dashed lines denote cutoffs. (**A**) CK–6H vs. SP–6H: 4 DEGs; (**B**) CK–6H vs. IND–6H: 33 DEGs; (**C**) CK–6H vs. SI–6H: 42 DEGs; (**D**) CK–24H vs. SP–24H: 687 DEGs; (**E**) CK–24H vs. IND–24H: 6 DEGs; (**F**) CK–24H vs. SI–24H: 273 DEGs.

**Figure 4:**
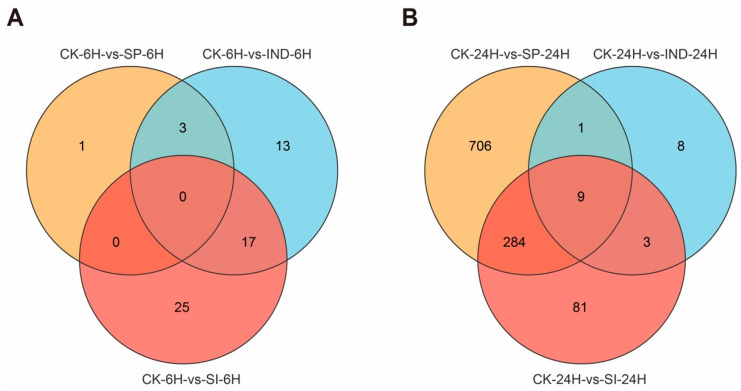
(**A**) Venn diagram showing differentially expressed genes (DEGs) from pairwise comparisons of CK versus SP, IND and SI samples under 6 h treatment; (**B**) Venn diagram showing differentially expressed genes (DEGs) from pairwise comparisons of CK versus SP, IND and SI samples under 24 h treatment. The numbers in each section indicate the quantity of unique or shared DEGs for the respective regions.

Venn diagram analysis was used to identify overlapping and unique DEGs among the three treatment groups at 6 h and 24 h (Figure 3). At 6 h, a total of 79 DEGs were identified across the SP, IND and SI groups, with no common DEGs shared by all three groups, indicating that the early transcriptional responses to single and combined insecticide exposure were highly specific (Figure 4). At 24 h, nine common DEGs were identified among all three treatment groups (Table 5), and the SI group had the largest number of unique DEGs, further confirming that the combined treatment triggered a distinct and extensive transcriptional cascade compared to single-agent exposure.

Functional annotation of the DEGs at 6 h showed enrichment of genes associated with detoxification metabolism (cytochrome P450s, carboxylesterases, glutathione S-transferases (GSTs), glycosyltransferases), transmembrane transport (ABC transporters), signal transduction (protein kinases) and innate immunity (antimicrobial peptides, lysosomal components). At 24 h, the DEG functional spectrum shifted significantly, with enrichment of genes related to lipid metabolism (phosphatidate phosphatase, sphingomyelin phosphodiesterase), carbohydrate and energy metabolism (glycogenin), and cellular degradation processes (lysosomal enzymes, autophagy-related genes), suggesting a transition from early defensive responses to late cellular damage and dysfunction in the SI treatment group. No differentially expressed genes were shared between the 6 h and 24 h time points in the SI treatment.

### 3.4. GO and KEGG Enrichment Analysis of DEGs Reveals Time-Dependent Functional Pathways in the Synergistic Response

#### 3.4.1. Early Response (6 h): Activation of Detoxification, Stress Signaling and Apoptosis Pathways by SI Treatment

**Figure 5:**
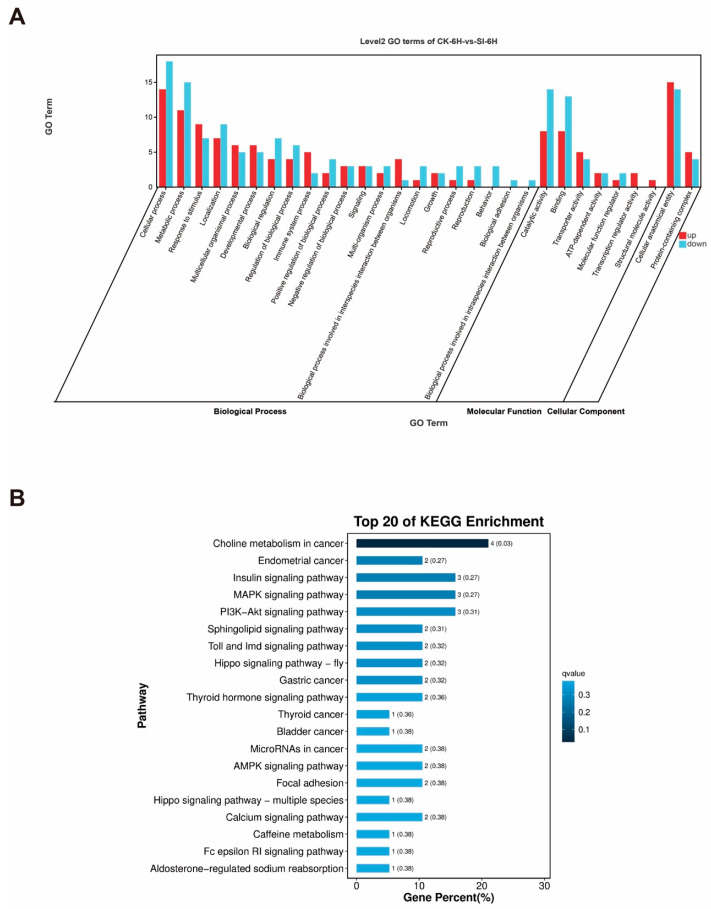
GO and KEGG enrichment analyses of differentially expressed genes between treatment groups at 6 h after exposure to the spinosad and indoxacarb combination. (**A**) GO annotations are categorized into three major functional groups: Biological Process, Cellular Component, and Molecular Function, showing the annotation terms and the corresponding percentages and numbers of genes in each functional category. (**B**) The Y-axis represents KEGG pathways, and the X-axis represents the gene ratio. The color of the dots indicates the q-value, and the dot size reflects the number of differentially expressed genes associated with the pathway.

GO enrichment analysis of DEGs in the SI vs. CK groups at 6 h (Figure 5A) showed that upregulated DEGs were annotated to 21 subcategories of Biological Process (BP), two subcategories of Cellular Component (CC) and six subcategories of Molecular Function (MF). The main enriched BP terms included metabolic process, cellular response to stimulus and biological regulation; CC terms were dominated by membrane and membrane part; MF terms included catalytic activity and binding, consistent with the early activation of detoxification and stress response processes.

KEGG pathway enrichment analysis (Figure 5B) revealed that the DEGs in the SI group at 6 h were significantly enriched in three major functional pathways: detoxification and metabolic competition pathways, cellular signal transduction pathways (insulin signaling pathway, MAPK signaling pathway, PI3K-Akt signaling pathway, calcium signaling pathway) and apoptosis-related pathways. Notably, the SI treatment uniquely activated the insulin and MAPK stress signaling pathways at 6 h—pathways not significantly enriched in the single-agent SP or IND groups—indicating that the combined treatment triggered an amplified early stress response in *C. medinalis* larvae.

#### 3.4.2. Late Response (24 h): SI Treatment Induces Activation of Autophagy–Lysosome, Lipid Metabolism and Neurodegenerative Pathways

**Figure 6:**
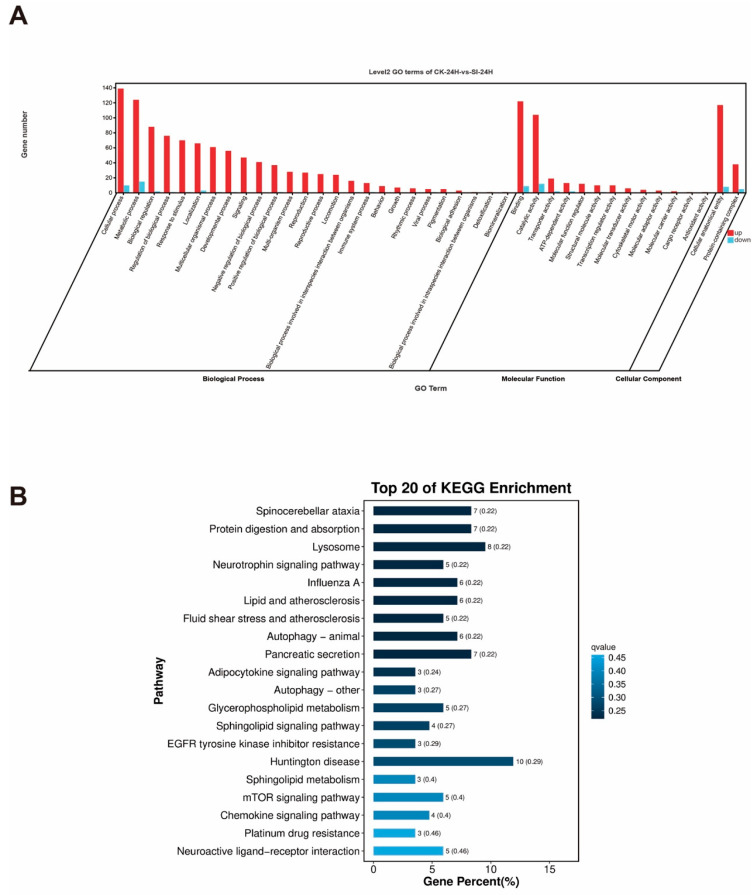
GO and KEGG enrichment analyses of differentially expressed genes between treatment groups at 24 h after exposure to the spinosad and indoxacarb combination. (**A**) GO annotations are categorized into three major functional groups: Biological Process, Cellular Component, and Molecular Function, showing the annotation terms and the corresponding percentages and numbers of genes in each functional category. (**B**) The Y-axis represents KEGG pathways, and the X-axis represents the gene ratio. The color of the dots indicates the q-value, and the dot size reflects the number of differentially expressed genes associated with the pathway.

At 24 h post-exposure, GO enrichment analysis of DEGs in the SI vs. CK groups (Figure 6A) showed a substantial expansion of annotated functional categories: upregulated DEGs were mapped to 26 BP subcategories, two CC subcategories and 13 MF subcategories. The BP terms were enriched in cellular process, metabolic process and response to stimulus; MF terms were further expanded to include hydrolase activity, transferase activity and transporter activity, reflecting extensive cellular metabolic and structural remodeling induced by the SI combination treatment.

KEGG pathway enrichment analysis (Figure 6B) identified the core pathways underlying the synergistic effect of the SI mixture at 24 h, which were distinctly different from the 6 h response and single-agent treatments: (1) autophagy–lysosome system: Significantly enriched in lysosome and autophagy—animal pathways, with key genes (ATG2B, Wipi2, Cp1, PSAP) significantly upregulated; (2) lipid metabolism disorders: Enriched in glycerophospholipid metabolism and sphingolipid metabolism pathways, with lipid metabolism-related genes (PNPLA8, SMSr) highly expressed; (3) neurodegenerative and neurotoxic pathways: Enriched in Huntington disease and spinocerebellar ataxia pathways—although these are human disease terms, in insects such enrichment typically reflects conserved stress responses (e.g., protein aggregation, mitochondrial dysfunction, or neuronal stress), indicative of neuronal stress and damage; and (4) digestive system damage: Enriched in protein digestion and absorption and pancreatic secretion pathways, suggesting severe midgut tissue injury.

None of these pathways were significantly enriched in the single-agent SP or IND groups at 24 h, confirming that the late-stage multi-pathway cellular damage was a unique transcriptional signature of the spinosad–indoxacarb synergistic combination.

## 4. Discussion

Pesticide mixture formulation has become a cornerstone strategy for mitigating insecticide resistance and improving control efficacy in integrated pest management (IPM) systems, particularly for highly destructive migratory pests such as *Cnaphalocrocis medinalis* that have evolved widespread resistance to conventional single-mode-of-action insecticides. In this study, we identified that spinosad–indoxacarb mixtures at multiple ratios exhibit robust synergistic toxicity against *C. medinalis* neonates, while other combinations (e.g., spinetoram–abamectin, methoxyfenozide–quinalphos) show antagonistic effects. Through comparative transcriptomic analysis at 6 h and 24 h post-exposure, we uncovered a dynamic, two-stage molecular mechanism underlying the spinosad–indoxacarb (SI) synergism: early potential competitive inhibition suggested by transcriptomic data of the detoxification system amplifies neurotoxicity, and late overactivation of autophagy–lysosomal pathways, coupled with lipid metabolism disruption and midgut damage, is associated with autophagy–lysosomal activation and cellular damage. This work provides the first transcriptomic evidence for the multi-pathway synergistic action of insecticide mixtures in *C. medinalis* and offers mechanistic insights into the rational design of pesticide formulations for resistance management.

A critical prerequisite for effective pesticide mixture design is the selection of combinations with distinct modes of action and non-overlapping metabolic detoxification pathways [11]. Our screening results showed that spinosad–indoxacarb mixtures at 1:1, 2:3 and 1:5 ratios all had a co-toxicity coefficient (CTC) > 120, confirming stable synergism, whereas spinetoram–abamectin and methoxyfenozide–quinalphos mixtures exhibited antagonism (CTC < 80). This discrepancy in interaction effects is likely attributed to the metabolic and target-site compatibility of the components: spinosad (a macrocyclic lactone targeting nicotinic acetylcholine receptors) [16] and indoxacarb (an oxadiazine blocking sodium channels) act on distinct neural targets and rely on partially overlapping but non-competitive detoxification enzyme systems (P450s for spinosad oxidation, esterases for indoxacarb activation) [17]. In contrast, spinetoram and abamectin both belong to macrocyclic lactones with similar target sites and detoxification pathways, leading to competitive binding at molecular targets and metabolic saturation, which ultimately reduces combined efficacy [18]. Methoxyfenozide (a juvenile hormone analog) and quinalphos (an organophosphate) may exhibit metabolic antagonism, where one compound induces detoxification enzymes that accelerate the degradation of the other. For instance, organophosphates can inhibit key metabolic enzymes involved in juvenile hormone processing [19]. These results reinforce the principle that ratio optimization and mechanistic compatibility are equally critical for successful mixture formulation, beyond simple selection of compounds with different modes of action. It should be noted that some CTC values (e.g., 121) were only slightly above the threshold of 120. Such borderline values should be interpreted cautiously as weak synergism, and CTC values from different populations are not directly comparable in terms of synergy intensity due to differences in baseline susceptibility. Therefore, the CTC index is best used as a qualitative indicator within a given population. The YZ25 field population (moderately resistant to both spinosad and indoxacarb) showed stronger synergism (CTC = 182.1 at 1:5) than the SS (CTC = 121 at 1:1), possibly because constitutively elevated detoxification enzymes (P450s/esterases) in resistant populations create a larger enzyme pool, leading to greater competitive saturation and enhanced synergism. Thus, the spinosad–indoxacarb mixture may be resistance-dependent—more effective where single-agent efficacy has declined—supporting its strategic use in resistance-prone areas.

The early transcriptional response (6 h) to SI treatment revealed the core toxicokinetic basis of synergism: competitive saturation of key detoxification enzymes (cytochrome P450s, esterases, GSTs, ABC transporters) by the two insecticides impairs metabolic clearance and activation balance, leading to toxic accumulation of both active ingredients [20]. Single treatments with spinosad (SP) and indoxacarb (IND) both induced moderate upregulation of detoxification-related genes (e.g., *CYP6B7*, *LIPI*, *Abcb1a*), a typical defensive response of insects to xenobiotic stress [21,22]. However, SI treatment led to non-additive expression changes in these genes—some P450 genes highly induced by single SP/IND showed insignificant expression under SI exposure, a hallmark of substrate saturation in detoxification enzyme systems [23]. This competitive inhibition has two key consequences: first, P450 enzymes responsible for spinosad oxidation are occupied by indoxacarb or its metabolites, prolonging spinosad persistence in the hemolymph and enhancing its neurostimulatory toxicity; second, esterase activity required for indoxacarb activation to its toxic metabolite DCJW is compromised by spinosad, disrupting the balance between activation and degradation and leading to abnormal accumulation of both parent and active indoxacarb forms [24]. Similar competitive detoxification has been reported in organophosphate–pyrethroid mixtures, where esterase inhibition by organophosphates impairs pyrethroid hydrolysis and amplifies toxicity [12], validating that toxicokinetic interference is a conserved mechanism of pesticide synergism across insect species [25]. Synergistic interaction between targets: Spinosad activates nAChRs, while indoxacarb blocks voltage-gated sodium channels. Their simultaneous action converges on neural signaling: spinosad-induced hyperexcitation increases the sodium channel opening frequency, making channels more susceptible to indoxacarb blockade; conversely, sodium channel inhibition impairs repolarization, amplifying excitotoxicity [26]. This dual “excitation-blockade” trap compromises neuronal function beyond additive expectation and likely underlies the early activation of stress pathways (e.g., MAPK, Insulin) uniquely observed in the mixture group (Figure 4B) [27]. Thus, synergistic interaction at the neural circuit level complements the toxicokinetic mechanism described above.

In the present transcriptomic dataset, we did not detect significant differential expression of the nicotinic acetylcholine receptor (nAChR) subunits—particularly the alpha-6 subunit and its transcript variants—which are the primary targets of spinosad, nor of the voltage-gated sodium channel targeted by indoxacarb. This absence of mRNA changes is not unexpected and can be attributed to several factors. First, transcript levels do not always reflect functional protein changes: post-transcriptional regulation, translation efficiency, and protein modifications (e.g., phosphorylation, subunit assembly) may all influence the actual sensitivity of the targets. Second, the primary action sites of these insecticides are in the nervous system, whereas our transcriptome samples were prepared from whole larval homogenates; specific signals from neural tissues could be diluted by background expression from other tissues [28]. Third, the two time points chosen (6 h and 24 h) may not capture transient or oscillatory changes in target gene expression; the synergistic effect likely relies more on metabolic and cellular damage pathways than on sustained transcriptional upregulation of the targets themselves. Moreover, we cannot exclude the possibility that functional alterations in target proteins (e.g., conformational changes, altered ion channel gating dynamics) occur without changes in mRNA abundance [29]. Therefore, direct functional validation—such as Western blotting, immunohistochemistry, electrophysiological recordings, and RNA interference—is needed in future studies to further assess the actual contribution of nAChR α6 subunits and sodium channels to the spinosad–indoxacarb synergism.

In addition to detoxification system inhibition, SI treatment uniquely activated the insulin and MAPK stress signaling pathways at 6 h, a response not observed in single-agent groups. The insulin pathway (via *insr*) and MAPK pathway (via *MKNK2*) are central to cellular responses to energy depletion, oxidative stress and neural damage [30,31]; their co-activation under SI treatment reflects a synergistic amplification of neural stress caused by the dual action of spinosad (nicotinic receptor hyperexcitation) and indoxacarb (sodium channel blockage). Spinosad alone did not significantly activate the insulin pathway in our transcriptomic data, while indoxacarb alone had no effect on this pathway. The combined treatment exacerbated this stress signal, as the simultaneous disruption of two key neural signaling pathways overwhelmed the insect’s homeostatic regulatory capacity, laying the foundation for subsequent cellular damage. This early stress signal amplification is a critical toxicodynamic component of SI synergism, distinguishing it from the simple additive effects of single-agent neurotoxicity.

The late transcriptional response (24 h) to SI treatment revealed a fundamental shift from defensive stress to multi-pathway cellular dysfunction, characterized by the overactivation of autophagy–lysosomal pathways, disruption of lipid metabolism, induction of neurodegenerative-like signaling, and midgut damage—these pathways were not significantly enriched in single SP/IND groups, representing a unique transcriptional signature of SI synergism. Autophagy is a conserved cellular protective mechanism that clears damaged organelles and proteins in response to stress [32], but SI treatment triggered its overactivation (via upregulation of *ATG2B*, *Wipi2*, *Cp1*, *PSAP*), suggesting a potential transition from protective autophagy to autophagy-associated cellular dysfunction [33]. The continuous induction of autophagy-related genes under SI exposure indicates that the rate of insecticide-induced cellular damage (e.g., mitochondrial dysfunction, protein misfolding) far exceeds the repair capacity of the autophagic system, ultimately depleting cytoplasmic resources, which may be associated with cell death. Concurrently, enrichment of the lysosome pathway suggests lysosomal membrane permeabilization (LMP), where hydrolytic enzymes such as cathepsins leak into the cytoplasm and directly induce cellular necrosis [34]—a phenomenon previously reported in insect cells exposed to insecticides, where LMP is a key mediator of cellular damage [35]. The combined overactivation of autophagy and lysosomal dysfunction thus correlates with late-stage SI toxicity.

Among the nine common DEGs upregulated by all treatments at 24 h (Table 5), PNPLA8 (phospholipase A2) and SMSr (sphingomyelin synthase) are directly involved in lipid metabolism and membrane homeostasis, while MKNK2 functions in stress signaling. Their consistent upregulation suggests a core transcriptional response to xenobiotic stress, although functional validation is needed. Disruption of lipid metabolism via upregulation of PNPLA8 and SMSr at 24 h further exacerbates the synergistic effect by impairing structural and physiological homeostasis of *C. medinalis* larvae. Lipid metabolism is critical for maintaining cell membrane integrity and fluidity—key for the function of neural ion channels (indoxacarb’s target) and receptor proteins (spinosad’s target) [36]. Upregulation of phospholipases (*PNPLA8*) and sphingomyelin synthases (*SMSr*) under SI treatment disrupts glycerophospholipid and sphingolipid metabolism, leading to cell membrane destabilization and further amplification of neural signal transduction defects [37]. Enrichment of protein digestion and absorption and pancreatic secretion pathways indicates severe midgut damage, a critical consequence given that the midgut is the primary organ for insecticide absorption, xenobiotic metabolism and nutrient acquisition in lepidopteran larvae [38]. Midgut cell damage not only interrupts energy supply (depleting ATP required for detoxification and stress response) but also compromises the intestinal barrier, allowing more insecticide to enter the hemolymph and amplify systemic toxicity [39]. This dual impairment of neural function and midgut physiology creates a positive feedback loop of toxicity, explaining why SI treatment leads to faster and more complete mortality than single-agent exposure.

From a resistance management perspective, our findings provide three key practical implications for the field application of spinosad–indoxacarb mixtures against *C. medinalis*. First, the key genes identified in this study—CYP6 family P450s, ABC transporters, and autophagy-related genes (*ATG2B*, *Wipi2*)—can serve as molecular markers for resistance monitoring. Quantitative detection of the expression levels of these genes in field populations can early predict the development of resistance to the SI mixture, enabling timely adjustment of IPM strategies. Second, prolonged continuous use of the SI mixture may select for field populations with enhanced broad-spectrum detoxification capacity (e.g., overexpression of P450s and ABC transporters that metabolize both spinosad and indoxacarb). Thus, rotational application with insecticides relying on distinct detoxification pathways (e.g., GST-dependent or hydrolase-dependent compounds) is recommended to reduce directional selection pressure. Third, the cellular-level endpoints identified (autophagy–lysosome activation, lipid metabolism disruption) can be used as screening criteria for new synergistic formulations, enabling rapid identification of true synergism (rather than simple additive effects) and accelerating the development of effective pesticide mixtures [40].

A notable finding of this study is the dose-ratio dependence of synergism, with spinosad–indoxacarb mixtures at 1:1, 2:3 and 1:5 ratios all exhibiting synergism (CTC > 120), and the 1:5 ratio showing the most pronounced effect (CTC = 182.1) in the YZ25 field population. This suggests that the competitive efficiency of the two insecticides for detoxification enzymes is adjustable via ratio optimization, and field populations with moderate resistance to single spinosad/indoxacarb may require specific ratios to achieve maximum synergism. Future research should further explore the impact of different spinosad–indoxacarb ratios on detoxification enzyme competition and cellular damage pathways, to develop population-specific formulation ratios for different rice-growing regions with varying resistance levels of *C. medinalis* [41].

This study has several limitations that warrant further investigation. First, the transcriptomic analysis identified key genes and pathways underlying SI synergism, but the functional validation of these genes (e.g., *CYP6B7*, *ATG2B*, *PNPLA8*) remains to be performed—including qRT-PCR confirmation of expression changes, as well as functional studies such as RNA interference (RNAi) or CRISPR-Cas9-mediated gene knockout to test whether silencing these genes abrogates the synergistic effect [42]. Second, this study focused on transcriptional changes, and metabolomic and proteomic analyses are needed to complement the molecular mechanism, by identifying changes in endogenous metabolites (e.g., neurotransmitters, lipid species) and protein expression that drive the observed phenotypic synergism. Third, the laboratory-based transcriptomic results need to be validated under field conditions, to evaluate the consistency of the synergistic mechanism and the real-world control efficacy of the SI mixture against natural *C. medinalis* populations [43]. Fourth, the current study tested only three mixture ratios (1:1, 2:3, 1:5); a wider gradient of ratios would be required to determine the absolute optimal synergistic ratio for field application. Additionally, the use of only two time points (6 h and 24 h) does not capture the full continuous transcriptional dynamics; future time-course studies with intermediate sampling (e.g., 12 h) are needed [44].

In conclusion, this study elucidates a dynamic, two-stage molecular mechanism for the synergistic effect of spinosad–indoxacarb mixtures against *C. medinalis*: early competitive inhibition of the detoxification system and amplification of stress signaling pathways prolong insecticide persistence and enhance neurotoxicity, while late overactivation of autophagy–lysosomal pathways, lipid metabolism disruption, and midgut damage lead to cellular damage [26]. This mechanistic framework not only explains the stable synergism of the SI mixture across multiple ratios but also provides a cellular and molecular basis for the rational design of pesticide mixtures for lepidopteran pest control [45]. The identified molecular markers and screening criteria can facilitate resistance monitoring and the development of new synergistic formulations, ultimately contributing to the sustainable management of *C. medinalis* and other insecticide-resistant agricultural pests [46].

## 5. Conclusions

In summary, we screened binary insecticide mixtures against *C. medinalis* and identified that spinosad–indoxacarb combinations exhibit stable synergism. Through comparative transcriptomic analysis, we revealed a dynamic two-stage mechanism: early competitive inhibition of detoxification enzymes and activation of stress signaling pathways amplify neurotoxicity, while late overactivation of the autophagy–lysosomal system, disruption of lipid metabolism, and midgut damage are associated with autophagy–lysosomal activation and cellular damage. These results provide the first transcriptomic evidence for the synergistic mechanism of this mixture and offer molecular markers and pathway-level insights to guide rational formulation design and resistance management for this major rice pest. Functional validation of the key genes identified here (e.g., qRT-PCR, RNAi) is currently ongoing and will be reported in future studies.

## Figures and Tables

**Table 1:** Joint toxicity of spinetoram, emamectin benzoate, and abamectin against newly hatched larvae of *C. medinalis.*

Insecticide	Population	Ratio	Slope	LC_50_ (95%FL) mg/L	ATI	TTI	CTC	Evaluation
spinetoram	LZ24	single	1.980 ± 0.301	0.0203 (0.0107–0.0333)	-	-	-	-
abamectin	LZ24	single	2.007 ± 0.286	0.213 (0.155–0.274)	-	-	-	-
emamectin benzoate	LZ24	single	2.103 ± 0.359	0.117 (0.053–0.220)	-	-	-	-
spinetoram:abamectin	LZ24	1:5	3.831 ± 0.874	0.1408 (0.0972–0.1745)	14.4	24.2	59.5	antagonism
spinetoram:emamectin benzoate	LZ24	1:10	2.012 ± 0.428	0.1433 (0.0906–0.1992)	14.1	24.79	56.85	antagonism

**Table 2:** Joint toxicity of spinosad and metaflumizone against newly hatched larvae of *C. medinalis.*

Insecticide	Population	Ratio	Slope	LC_50_ (95%FL) mg/L	ATI	TTI	CTC	Evaluation
spinosad	LZ24	single	2.699 ± 0.494	0.100 (0.067–0.129)	-	-	-	-
metaflumizone	LZ24	single	2.549 ± 0.372	0.067 (0.050–0.084)	-	-	-	-
spinosad:metaflumizone	LZ24	1:1	1.961 ± 0.335	0.1688 (0.0532–0.3034)	59.2	50.745	116	additive effect
spinosad:metaflumizone	LZ24	2:1	1.961 ± 0.335	0.0211 (0.00665–0.03792)	317.5	77.89	408	synergism

**Table 3:** Joint toxicity of spinosad and indoxacarb against newly hatched larvae of *C. medinalis.*

Insecticide	Population	Ratio	Slope	LC_50_ (95%FL) mg/L	ATI	TTI	CTC	Evaluation
indoxacarb	SS	single	2.501 ± 0.357	0.151 (0.122~0.188)	-	-	-	-
spinosad	SS	single	2.699 ± 0.494	0.044 (0.037–0.129)	-	-	-	-
indoxacarb	YZ25	single	1.786 ± 0.410	0.187 (0.100–0.281)	-	-	-	-
spinosad	YZ25	single	3.158 ± 0.629	0.132 (0.086–0.173)	-	-	-	-
spinosad:indoxacarb	SS	1:1	2.580 ± 0.604	0.056 (0.031–0.077)	78.6	64.55	121	synergism
spinosad:indoxacarb	SS	2:3	2.982 ± 0.771	0.062 (0.033–0.082)	71	57.46	123	synergism
spinosad:indoxacarb	YZ25	1:5	2.024 ± 0.408	0.096 (0.055–0.137)	137.5	75.5	182.1	synergism

**Table 4:** Joint toxicity of methoxyfenozide and quinalphos against neonate larvae of *C. medinalis.*

Insecticide	Population	Ratio	Slope	LC_50_ (95%FL) mg/L	ATI	TTI	CTC	Evaluation
Methoxyfenozide	YZ25	single	1.432 ± 0.327	4.217 (2.631–7.253)	-	-	-	-
Quinalphos	YZ25	single	1.603 ± 0.390	11.353 (7.165–18.675)	-	-	-	-
Methoxyfenozide:Quinalphos	YZ25	1:1	1.675 ± 0.379	6.397 (4.185–10.017)	65.92	68.57	96.1	antagonism
Methoxyfenozide:Quinalphos	YZ25	1:2	1.584 ± 0.370	6.914 (4.453–11.476)	60.99	79.05	77.2	antagonism

**Table 5:** Common differentially expressed genes at 24 h under single and mixed insecticide treatments.

Gene ID	TPM (Average)				Log2 Fold Change			Description
	CK	SP	IND	SI	CK vs. SP	CK vs. IND	CK vs. SI	
Cmed128380	1.69	12.29	1.91	9.24	2.87	0.18	2.45	XP_028160052.1 uncharacterized protein LOC114352599 isoform X1 [*Ostrinia furnacalis*]
Cmed049150	7.49	32.22	6.33	26.18	2.10	−0.24	1.80	XP_026316138.1 calcium-independent phospholipase A2-gamma-like [*Hyposmocoma kahamanoa*]
Cmed033240	7.53	21.65	9.37	25.86	1.52	0.02	1.78	XP_028159490.1 multiple C2 and transmembrane domain-containing protein-like [*Ostrinia furnacalis*]
Cmed127200	5.18	18.99	5.46	16.58	1.87	0.07	1.68	RVE43550.1 hypothetical protein evm_011777 [*Chilo suppressalis*]
Cmed023430	7.53	27.77	9.21	23.66	1.88	0.29	1.65	KAG6443452.1 hypothetical protein O3G_MSEX002873 [*Manduca sexta*]
Cmed096670	7.16	25.75	8.47	21.80	1.85	0.24	1.61	XP_028164690.1 vanin-like protein 1 isoform X1 [*Ostrinia furnacalis*]
Cmed029680	37.68	91.26	39.73	89.81	1.28	0.076	1.25	XP_026751274.1 MAP kinase-interacting serine/threonine-protein kinase 1-like [*Galleria mellonella*]
Cmed093030	24.18	55.67	20.64	48.53	1.20	−0.23	1.01	XP_028161084.1 sphingomyelin synthase-related 1-like [*Ostrinia furnacalis*]
Cmed063320	225.17	99.06	247.77	63.99	−1.18	0.14	−1.82	XP_028172899.1 vanin-like protein 1 [*Ostrinia furnacalis*]

## Data Availability

Data is contained within the article.
